# Retracted: The Design and Implementation of Postprocessing for Depth Map on Real-Time Extraction System

**DOI:** 10.1155/2017/4583434

**Published:** 2017-07-16

**Authors:** The Scientific World Journal

The Scientific World Journal has retracted the article titled “The Design and Implementation of Postprocessing for Depth Map on Real-Time Extraction System” [1]. The peer review of the article has been compromised, due to a conflict of interest between the handling editor and one of the authors.
